# Tomato Flour

**Published:** 1883-06

**Authors:** 


### TOMATO FLOUR.

The Italians dry and pulverize the pulp of the tomato. Large districts are devoted to the culture of the fruit for this purpose, the plant being usually raised between rows of vines in vineyards for the sake of economy of land. The ripe fruit is macerated in water, and when reduced to a thin pulp is strained to take out the seeds, cores, etc., and then spread in the sun to dry. It is afterward ground and put up for market. There seems to be no reason why evaporating ovens, so much in use for drying less succulent fruit, as apples, might not be utilized in this country for preparing tomatoes by drying.

Of course powdered tomato might not supersede the canned fresh fruit. Its chief use would be for soups, sauces, and other auxiliary uses in cooking. But there are many consumers of the fresh tomato who refuse the tinned canned tomato from fear of the action of the acid of the fruit on the leaded tin of the can, the resultant being in their estimation a virulent lead poison. Tomatoes put up in glass —quite high priced—have therefore been welcomed by lovers of the fruit—or vegetable. Possibly there is room here for an addition to our list of dried or evaporated food articles.

